# Assessment of the healthcare burden of dengue disease in Germany: a retrospective analysis of statutory health insurance data (2014–23)

**DOI:** 10.1093/jtm/taag047

**Published:** 2026-06-11

**Authors:** Christoph Lübbert, Markus Frühwein, Carolin Stephanie Dinjer, Caroline Mächler, Eduardo Bittencourt de Gomensoro, Tobias Vogelmann, Bojana Milovanović

**Affiliations:** Division of Infectious Diseases and Tropical Medicine, Department of Medicine, Leipzig University Medical Center, Leipzig, Germany; Interdisciplinary Center for Infectious Diseases, Leipzig University Medical Center, Leipzig, Germany; Department of Infectious Diseases and Tropical Medicine, Hospital St. Georg gGmbH, Leipzig, Germany; Dr. Frühwein and Partner, Practice for General Medicine, Travel Medicine and Tropical Disease, Munich, Germany; Takeda Pharma Vertrieb GmbH & Co. KG, Berlin, Germany; Takeda Pharmaceuticals International AG, Zurich, Switzerland; Takeda Pharmaceuticals International AG, Zurich, Switzerland; LinkCare GmbH, Ludwigsburg, Germany; Takeda Pharmaceuticals International AG, Zurich, Switzerland

**Keywords:** Travel, Epidemiology

## Abstract

**Background:**

The burden of dengue disease in Germany is a growing public health concern. While travellers to dengue-endemic countries are at increased risk of contracting a dengue infection, data describing dengue-related healthcare utilization and outcomes in Germany are limited. This study investigated the healthcare burden of dengue disease in travellers returning to Germany.

**Methods:**

A retrospective analysis (InGef research database) was used to estimate dengue incidence using anonymized representative data from the German Statutory Health Insurance from approximately 7 million insured persons/year during 2015–23. Outpatient and inpatient healthcare use and dengue associated costs were investigated.

**Results:**

From 2015 to 2023, among 10 151 240 insured persons, 887 dengue episodes were recorded. Patients had a median age of 33 years (interquartile range 26–46) and 52% were male. Each year, 103–182 episodes were reported (COVID-19 pandemic period [2020–22]: 10–62). Excluding the pandemic years, incidence was 1.4–2.5/100 000 persons, with an estimated total of 1453–2639 cases/year in Germany. Of the 37% hospitalized persons, 4% required intensive care. Mean hospital stay was 3.3 days; most patients were discharged after 1 overnight stay and no dengue related deaths were reported. Overall, 41% of patients took sick leave during their episode (mean sick leave for hospitalized patients: 8.8 working days); 38% of those not hospitalized took sick leave lasting 6.9 working days on average. Patients with dengue had 2-fold increased healthcare visits, resulting in a 162% increase in costs (mean costs/episode/quarter: 930€) compared with the pre-index period, with these costs mainly driven by outpatient care (23%) and hospitalization (64%). Hospitalized patients had a 4-fold rise in costs (quarterly costs/episode: 1700€; inpatient care accounting for 80%) compared with the pre-index period.

**Conclusions:**

Dengue virus infection in travellers returning to Germany impacted the healthcare system in Germany, leading to high costs, hospitalizations and physician visits and considerable sick leave.

## Introduction

Dengue is a mosquito-borne tropical disease caused by dengue virus (DENV) infection, leading to mild, flu-like symptoms in most cases; however, some patients develop severe disease, with potentially fatal bleeding and organ damage.^1^ Fatigue may also occur weeks or months after infection.^1^^,^^2^ There are four different DENV serotypes; infection with one serotype leads to long-term serospecific immunity against the corresponding serotype and only temporary immunity against the other serotypes.^3^ As there is no specific treatment for dengue, the focus of clinicians is symptom management.^4^ Dengue cases are classified into severe and non-severe cases.^4^ Worldwide, 136 countries or territories have documented transmission of dengue. In 2024, the World Health Organization (WHO) received reports of over 14.2 million dengue cases and over 10 000 fatalities.^3^

Dengue virus is transmitted primarily by the bite of a female *Aedes aegypti* mosquito, which is endemic in tropical and subtropical regions but is spreading geographically; this poses a risk of dengue infection for travellers visiting endemic countries. The Asian tiger mosquito, *Aedes albopictus*, is a secondary, increasingly important vector of dengue and has become established in several European countries, including Germany, increasing the risk of dengue transmission in the region.^4^ In Germany, the presence of *A. albopictus* has been recorded in parts of Baden-Württemberg and the Rhine-Main region (Rhineland-Palatinate, Hesse), downstream along the Rhine in Bonn (North Rhine-Westphalia) and in individual cities in Bavaria, Thuringia, as well as Berlin.^5^

The incidence and disease burden of dengue in travellers from endemic to non-endemic countries such as Germany is uncertain due to underreporting or inconsistent reporting. In line with the global increase in dengue cases, dengue cases in Germany increased in the period from 2014 to 2023, with the highest number of yearly cases (1717) recorded among travellers in 2024.^6^

Dengue has been classified as a notifiable disease in Germany since 2001 and national data are collected via the Robert Koch Institute (RKI); the Infectious Epidemiological Yearbook of Notifiable Diseases published by the RKI provides information on the number of dengue cases reported yearly in Germany. In 2019, 5.9 million long-distance trips were made from Germany, of which 18% were to Southeast Asia, 11% to South America, 11% to the Caribbean and 8% to India.^7^ In that year the RKI published that 1176 dengue cases were reported (including three cases with haemorrhagic fever), with 34% of cases leading to hospitalization.^8^ There were no recorded deaths and the incidence was 1.4 cases per 100 000 inhabitants.^8^ Dengue was most frequently contracted in Thailand (31%), followed by Indonesia (8%), India and Cuba (6% each) and Mexico (5%).^8^ The highest incidence was observed in the 20 to 39 age group, with both sexes equally affected.^8^ There is, however, a lack of information on outpatient care and several aspects of inpatient care in Germany, which include length of hospital stay and therapies or medications used.

More detailed information on inpatient care is available from the Institute for the Hospital Remuneration System (InEK); in 2019, 355 dengue cases were treated as inpatients in Germany and the average length of hospital stay was 4.3 days.^9^ As the classification is based on documented ICD-10 codes and not reported information, the InEK data are not fully comparable with the data from the RKI; this makes it difficult to obtain comparative statements regarding the burden of dengue disease. Additionally, routinely collected information on outpatient care cannot be generated from either data source, as RKI data rely on active reporting and InEK data cover hospital data only.

The availability of data on the impact of dengue on healthcare utilization in Germany can potentially help healthcare providers, travellers and public health bodies understand the true impact of dengue and its consequences in the non-dengue endemic, European environment. Therefore, a retrospective analysis of representative data from the German Statutory Health Insurance (SHI) for the years 2014 to 2023 was carried out to make comprehensive statements on the incidence of dengue disease and associated hospitalizations in Germany and to be able to describe the disease burden more comprehensively. In addition to information on sex and age, the data also contain information on medically diagnosed diseases and the services used by the SHI system,^10^ which makes it possible to describe both the incidence and the disease burden of dengue patients.

## Methods

### Objectives

The primary objectives of this study were as follows:

- Estimate the proportion of hospitalized dengue infections and describe its trend in Germany between 2014 and 2023
- Estimate the outpatient/inpatient health resource use and cost associated with dengue (with and without hospitalization) between 2015 and 2023

The secondary objectives of this study were as follows:

- Describe the demographic and clinical characteristics of patients hospitalized with dengue in Germany
- Describe and estimate the proportion of predefined complications in patients hospitalized with dengue
- Estimate all-cause mortality proportions for patients hospitalized with dengue in Germany

### Study setting and participant selection criteria

This is a retrospective, observational study with longitudinal analyses. An anonymized SHI database (Institut für angewandte Gesundheitsforschung Berlin GmbH (InGef)) was used to identify episodes of dengue diseases (defined by ICD-10 code) occurring between 1 January 2014, and 30 September 2023. In Germany, about 85% of German inhabitants are covered by SHIs, while the InGef sample database (consisting of 5% of the German population) is drawn to ensure representativeness and validity of the database used for health services research.^11^ Within the index period, each dengue disease episode was assigned an individual index quarter in which the diagnosis of dengue disease was coded. To describe the defined outcomes, the respective dengue disease episodes were followed up for 3 months. The follow-up period began after the index quarter and a pre-index was used to monitor dengue cases according to certain criteria (e.g. complications); the pre-index was defined as 1 year before the index quarter (1 January 2014 to 31 January 2014). Cases that took place during this period were included in the study of incidence but were excluded from other outcome measures.

All individual dengue disease episodes in the index period 1 January 2015, to 30 September 2023, per year were selected, based on following definitions:

- A corresponding ICD-10 diagnosis (ICD-10 codes A90, A91, A97 [and all underlying codes])
- No ICD-10 code of dengue during the 12 months prior to the index quarter (pre-index) to select incident dengue periods

After selection of dengue disease episodes, periods with dengue-associated hospitalizations were assigned. A dengue disease episode with associated hospitalization was defined as:

- Dengue disease with hospitalization during the index quarter or the quarter after the index quarter
- Dengue disease as the main or secondary diagnosis (ICD-10 code A90, A91, A97 [and all underlying codes])

Since a person can contract dengue multiple times due to four different DENV serotypes, each dengue episode was counted separately. In addition, the total population of all dengue episodes was stratified into periods with and without hospitalization.

### Variables

#### Exposures

The study population of interest for the main objectives of this study is patients with dengue disease, stratified into patients with and without hospitalization.

#### Outcomes and endpoints

To determine the incidence of dengue, individual patients and the number of periods with dengue were divided by the total number of insured individuals in the database. To determine the proportion of dengue disease periods with/without hospitalization, the number of patients with/without hospitalization was divided by the number of dengue disease episodes.

To determine the proportion of the different care pathways, the number of dengue disease episodes of the respective care pathways were divided by the total number of dengue disease episodes.

Patient characteristics are described for the overall study population and dengue disease episodes with/without hospitalization (Supplementary Table S1). Complications outcomes (Supplementary Table S2) were analysed for the index quarter and the 3-month follow-up period; patients with at least 1 code for the defined outcome in the database were counted towards these analyses. The hospitalization figures were evaluated for all hospitalizations (all cause hospitalization) and for dengue-associated hospitalization (dengue = main inpatient diagnosis), see Supplementary Table S3. Sick leave days outcomes are documented in Supplementary Table S4. The all-cause costs were evaluated and then stratified by sector; the costs for hospitalized cases with severe courses were determined and compared with the average costs of the other dengue patient groups (with and without hospitalization), see Supplementary Table S5. The mortality in the index quarter and in the follow-up (3 months) was determined and deceased patients described in terms of age and comorbidity (Supplementary Table S6).

### Statistical analysis

Patient and clinical characteristics were analysed descriptively using measures including mean, standard deviation (SD), confidence intervals (CI), median, interquartile range (IQR), minimum and maximum values for continuous variables and numbers and proportions for categorical variables. Estimates were extrapolated to the whole German population, taking the limitations into account.

## Results

### Patient numbers and demographics

**Figure 1:**
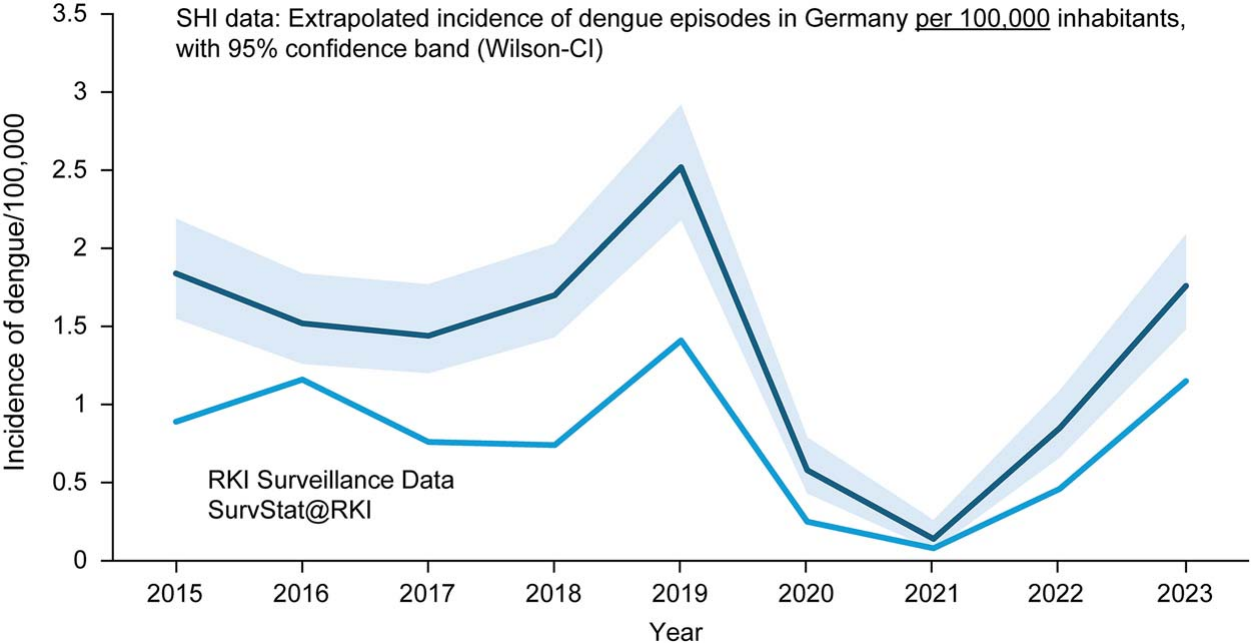
Dengue incidence in Germany between 2015 and 2023.

From 2015 to 2023, of the 10 151 240 insured individuals captured in the database, 887 dengue episodes were recorded: 329/887 (37%) dengue episodes resulted in hospitalization while 558/887 (63%) dengue episodes did not. Patients with dengue had a median age of 33 years (IQR 26–46) and 52% were male. The number of patients stratified by age and sex in the index quarter is shown by table 1. Each year, 103–182 episodes of dengue were reported (COVID-19 pandemic period [2020–22]: 10–62). Excluding the COVID-19 pandemic years, the extrapolated incidence of dengue episodes was 1.4–2.5/100 000 persons, with an estimated total number of 1453–2639 cases/year (Figure 1).

**Table 1:** Number of patients stratified by age and sex in the dengue disease episode (index quarter).

	Dengue patients						Without hospitalization						With hospitalization					
	Female		Male		Total		Female		Male		Total		Female		Male		Total	
*N* Patients total (reference value)	*N*	%	*N*	%	*N*	%	*N*	%	*N*	%	*N*	%	*N*	%	*N*	%	*N*	%
Age (in years)	418	100	457	100	875	100	262	100	285	100	547	100	156	100	172	100	328	100
<18	17	4.07	19	4.16	36	4.11	11	4.20	11	3.86	22	4.02	6	3.85	8	4.65	14	4.27
18–29	157	37.56	146	31.95	303	34.63	106	40.46	82	28.77	188	34.37	51	32.69	64	37.21	115	35.06
30–39	103	24.64	120	26.26	223	25.49	59	22.52	80	28.07	139	25.41	44	28.21	40	23.26	84	25.61
40–49	69	16.51	66	14.44	135	15.43	43	16.41	39	13.68	82	14.99	26	16.67	27	15.70	53	16.16
50–59	50	11.96	68	14.88	118	13.49	29	11.07	47	16.49	76	13.89	21	13.46	21	12.21	42	12.80
≥60	22	5.26	38	8.32	60	6.86	14	5.34	26	9.12	40	7.31	8	5.13	12	6.98	20	6.10
Mean age	35.3		37.0		36.2		34.9		37.7		36.3		36.0		35.7		35.9	
SD	13.6		14.4		14.0		13.9		14.3		14.2		13.2		14.4		13.9	
Min	6		2		2		6		5		5		7		2		2	
Max	94		88		94		94		88		94		70		75		75	
Q25	26		27		26		25		27		26		26		25		26	
Q50	32		33		33		31		35		33		33		32		33	
Q75	45		48		46		43		50		46		46		45		45	
Number of patients < 65 years	407	97.37	444	97.16	851	97.26	254	96.95	278	97.54	532	97.26	153	98.08	166	96.51	319	97.26
Insurance status																		
Member	321	76.79	384	84.03	705	80.57	197	75.19	241	84.56	438	80.07	124	79.49	143	83.14	267	81.40
Family insurance	75	17.94	56	12.25	131	14.97	50	19.08	34	11.93	84	15.36	25	16.03	22	12.79	47	14.33
Pensioner	22	5.26	17	3.72	39	4.46	15	5.73	10	3.51	25	4.57	7	4.49	7	4.07	14	4.27

If patients had two or more dengue episodes in the index period, only the first episode was used to describe age/sex/insurance status.

### Complications, hospitalization and comorbidities

In total, 37% of patients with dengue required hospitalization (Supplementary Figure S1), with 64% (*n* = 209/329) of hospitalized patients treated in university hospitals. Furthermore, 4% of all dengue episodes treated in hospitals were on the intensive care unit (ICU) (*n* = 12/329), which corresponds to 13 cases per year in Germany.

While the average hospital length of stay was 3.3 days, the median length of stay was 1 day, meaning that 50% of patients were discharged after a single overnight stay (mean sick leave of 46% of patients: 8.8 working days). Overall, 41% of patients took sick leave for 7.7 working days during their episode (38% of those not hospitalized took sick leave lasting 6.9 working days on average).

**Figure 2:**
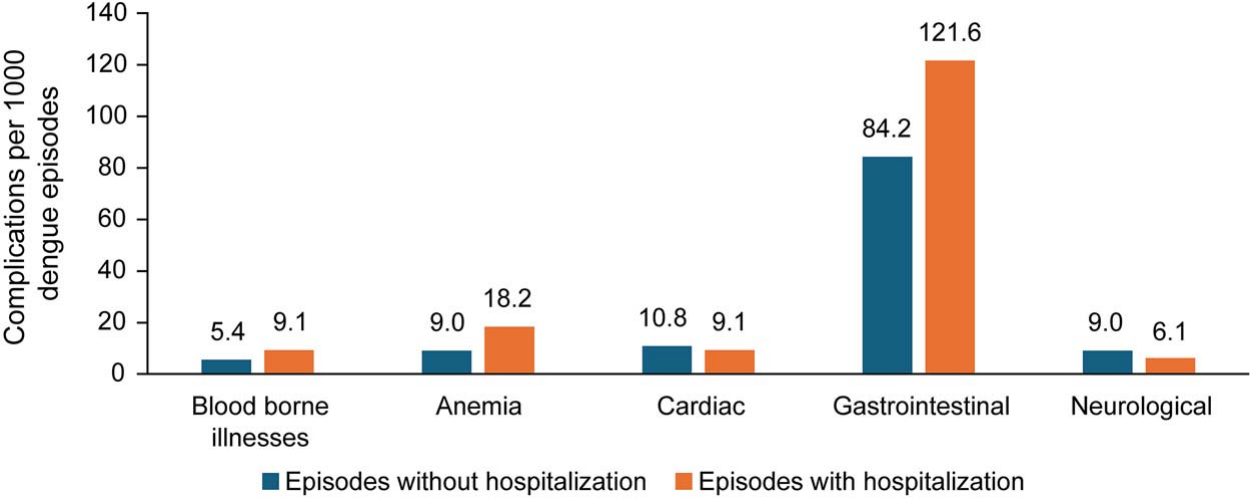
Complications for dengue episodes with (*n* = 329/887) and without (*n* = 558/887) hospitalization.

During the study period, 6 out of 875 patients died; however, none were related to dengue, or within the index or follow-up quarter. Episodes requiring hospitalization were twice as likely to be associated with complications, with gastrointestinal being the most common (Figure 2 and Supplementary Figure S2).

### Physician visits

**Figure 3:**
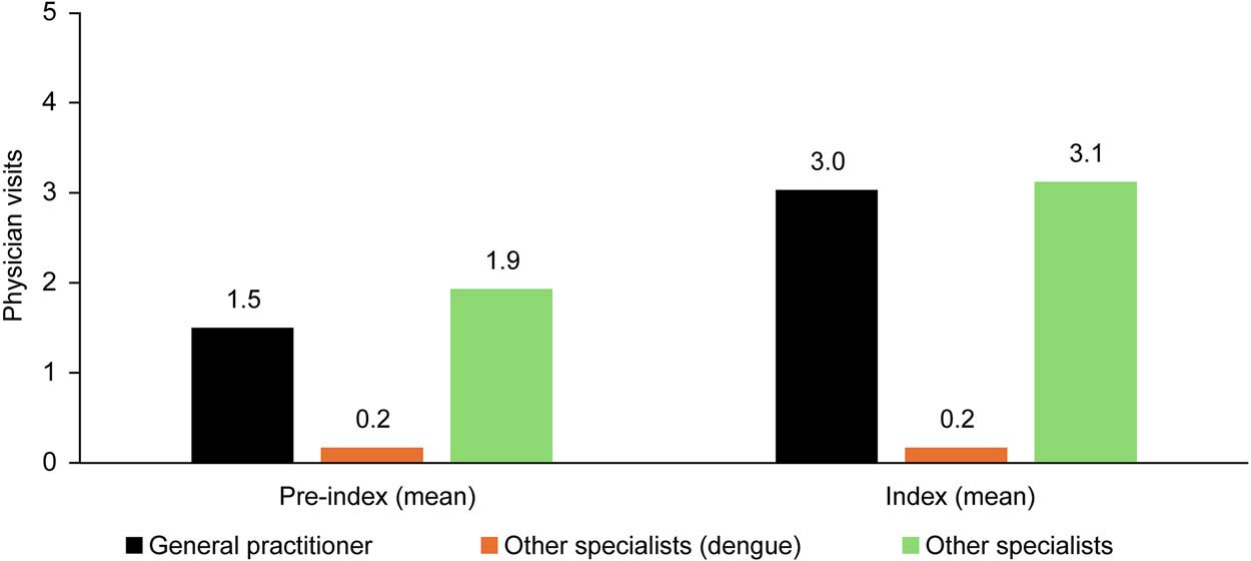
Physician visits during pre-index and index periods of patients with hospitalized dengue.

An increase in visits to both general practitioners and other specialists was observed during the index period compared with the pre-index period, with visits to specialists (identified as being capable of treating dengue) comparable across both periods (Figure 3). Patients with dengue doubled their physician visits, peaking at seven visits during the dengue quarter, then returning to baseline levels.

### Costs

**Figure 4:**
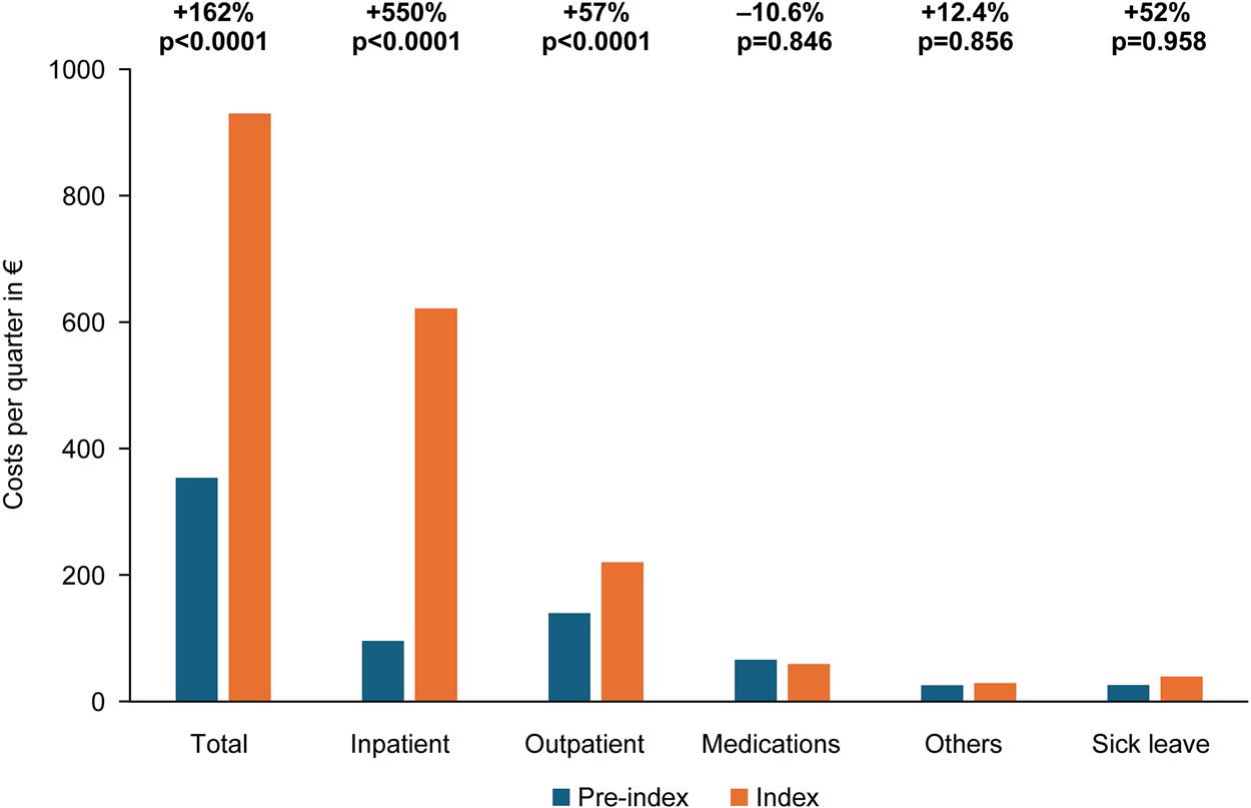
Overall and stratified costs associated with dengue.

Patients with dengue had 2-fold more healthcare visits, resulting in a 162% increase in costs (mean costs/episode/quarter: 930€) compared with the pre-index period (Figure 4). The rise in total costs during dengue was driven by a 5.5-fold increase in inpatient costs and an approximate 50% increase in outpatient costs. Post-dengue costs remained 40% higher than before in hospitalized dengue episodes.

Average costs rose by a factor of 1.6 to 930€ in the dengue quarter compared with the previous quarter, highlighting increased healthcare utilization (Supplementary Figure S3). During the dengue episode, costs of around 1000€ were driven by hospitalization (64%) and outpatient care (23%) (Supplementary Figure S4). Hospitalization episodes drove quarterly costs to 1700€, with inpatient care accounting for 80% (Supplementary Figure S5).

## Discussion

This study documents the health economic burden of dengue disease in Germany from the perspective of the German SHI, with episodes of dengue disease evaluated that occurred between 1 January 2015, and 30 September 2023. We found that 37% of patients with dengue required hospitalization; of hospitalized episodes, 64% were treated in university hospitals, with 4% requiring ICU care. During the dengue index quarter, every 10th patient suffered from complications, which were most likely coded as gastrointestinal. To alleviate symptoms of dengue, paracetamol may be prescribed, especially in the outpatient sector, but effective patient follow up remains particularly important.^3^

Average total costs per dengue episode amounted to 930€, a rise by a factor of 1.6 compared to the quarter before, highlighting increased healthcare utilization. Post-dengue costs remained 40% higher than before in hospitalized dengue episodes, possibly because the episode exceeded the index quarter. We also found that dengue patients doubled their physician visits, peaking at seven visits during the dengue quarter, then returning to baseline levels. The economic production loss associated with dengue primarily reflects sick leave covered by health insurance. Based on claims data, we estimate that around 500 symptomatic dengue cases per year could not have been reported to German health authorities, which suggests that the healthcare and economic burden of dengue virus infection in Germany is likely to be higher than previously reported.

A recent publication demonstrated the healthcare burden of dengue in Germany by comparing patients with dengue to those without dengue disease between 2015 and 2018.^12^ Compared to controls, dengue patients had significantly higher healthcare resource utilization, including 3.2 times higher probability of being hospitalized, 8.2 more outpatient visits and 9.5 additional sick leave days, with costs for total healthcare, inpatient care and outpatient care all increasing significantly. Furthermore, an international prospective study, which included Germany, found that the percentages of travellers that were hospitalized and received outpatient care due to dengue was higher after returning home, compared to the countries they returned from.^13^ Furthermore, a review of GeoSentinel surveillance records from 2007 to 2022 of dengue among travellers residing in multiple non-endemic countries, including Germany, found that 724 (26.7%) of patients with dengue were hospitalized.^14^ The most frequent regions of acquisition were Southeast Asia (50.4%), South-Central Asia (14.9%), the Caribbean (10.9%) and South America (9.2%).^14^ There were no dengue associated deaths in our study and reported fatal dengue infections in Germany are very rare in the literature; recent cases have involved German travellers who returned from trips to Ecuador,^15^ Guadeloupe,^16^ Sri Lanka,^17^ Thailand^18^ and Togo.^19^ The impact of dengue on the elderly should also be considered, an analysis from the global burden of disease study 2021 found that the dengue burden among adults aged 70 years and above has significantly increased between 1990 and 2021, with the highest burden primarily concentrated in Tropical Latin America, South Asia and Southeast Asia.^20^ While these data are not focused on travellers, it should be noted that substantial numbers of travellers above the age of 50 travel to these regions.^14^

Dengue incidence in Germany remains low compared to incidence in endemic countries and high-risk regions. The highest number of yearly cases in Germany (1717) was recorded among German travellers in 2024^6^; this trend is consistent with the global increase in dengue cases in the same year. Differentiated pre-travel advice and preventive measures are therefore becoming more important.^21^^,^^22^ The increasing spread of dengue beyond traditional endemic regions can be attributed to factors including climate change, globalization and urbanization; outbreaks in non-travellers in Germany could therefore be expected in the future.^23^

The impact of travel-related dengue on the economic and healthcare burden in Germany is expected to increase in the future. Within the upcoming 30 years, *A. albopictus* may have found suitable living conditions in about 68% of the European continent, including most of the British Isles, Ireland and southern Scandinavia, while about 83% of urban areas are expected to become suitable for *A. albopictus* in the future.^24^

There is a possibility that the high rate of gastrointestinal complications associated with DENV infection that we report is likely to be due, in part, to double infections involving, e.g., travellers’ diarrhoea. There is no specific data available on German travellers with double infections, but it should be noted that gastrointestinal tract coinfections frequently occur among patients with dengue in dengue endemic countries, with typhoid coinfections reported in 17%^25^ and 19%^26^ of patients with dengue in India and Nepal, respectively.

In addition to education for how travellers can best avoid insect bites, implementation of preventive measures such as vaccination should be considered.^27^^,^^28^

### Limitations

The study has several limitations, some of which relate to our use of the SHI database. The quality and accuracy of the data within the database relies on the completeness and correctness of the recorded information. With less than 2000 cases per year, dengue is a rare disease in Germany. Differentiating dengue from other febrile illnesses, especially in early stages, can be challenging for physicians leading to underreporting. Indirect costs associated with, e.g., production losses, were not covered, due to these costs being beyond the scope of the study. Furthermore, only billable services are recorded, which may lead to an underestimate of unbilled services.

Asymptomatic dengue cases and mild cases that did not receive medical treatment are not recorded in the database. This could lead to an overrepresentation of severe symptomatic dengue cases and a corresponding underrepresentation of asymptomatic or mild cases that were not diagnosed. Asymptomatic and mild primary infections, which are known or unknown to the infected person, are particularly important, as secondary dengue infections normally carry a higher risk of severe disease compared to primary infections.^1^ An added complication is that the database does not include cases that involved the receipt of medical treatment at the travel destination rather than Germany. One study found that among 201 foreign travellers hospitalized due to fever in the Indonesian island of Bali, dengue was confirmed in 133 (66.2%) of them^29^; interestingly, the majority (59.7%) of them had a primary infection.^29^

Coding limitations or errors in data entry may lead to misclassification and miscoding; data may be subject to administrative and reporting bias due to the retrospective nature of the study, which relied on diagnostic codes without laboratory confirmation. The cases recorded in the SHI databases relate only to individuals with insurance coverage. The data do not include privately insured patients (approximately 10% of the population) with a higher average socioeconomic status, which in turn is associated with more long-distance travel. Finally, this study includes cases during the COVID-19 pandemic (2020–22), and care should be taken when interpreting the number of recorded dengue cases during this period. While dengue cases in Germany declined significantly during the pandemic, this is mainly due to the introduction of extensive travel restrictions to contain the spread of COVID-19.

## Conclusions

In our study, travel-related dengue cases impacted the healthcare system in Germany, leading to high costs, hospitalizations and physician visits, as well as sick leave. Given the global increase in dengue cases combined with an increasing number of cases in Germany, the study findings demonstrate a need for targeted public health interventions for travellers going to and coming from endemic regions and preventive measures to mitigate the impact of dengue on the German healthcare system.

## Supplementary Material

Supplementary_material_taag047
