# Supplementary material for: Assessment of the healthcare burden of dengue disease in Germany: a retrospective analysis of statutory health insurance data (2014–23)
Source: J Travel Med. 2026 Jun 11;33(5):taag047. doi: 10.1093/jtm/taag047 (PMC13318845; doi:10.1093/jtm/taag047)
Supplement: Supplementary_material_taag047 [file supplementary_material_taag047.zip › BOD+in+germanyMS_supplementary_22May2026.docx]

**Supplementary figures**

**Supplementary Figure 1: Proportion of dengue episodes that required hospitalization.** SHI, Statutory Health Insurance; RKI, Robert Koch Institute

**Supplementary Figure 2: Complications recorded per 1000 dengue episodes in the quarter before, after and during dengue diagnosis.**

**Supplementary Figure 3:** **Average costs in the pre-index and index quarters**

**Supplementary Figure 4:** **Total and stratified costs of dengue episodes**. N= 887 episodes.

**Supplementary Figure 5: Total and stratified costs of hospitalized dengue episodes. N=329 episodes.**

**Supplementary tables**

**Supplementary Table 1: Patient characteristics according to the index quarter**

| **Endpoint** | **Output** |
| --- | --- |
| Age | Ø, SD, Min, Max, Q25, Q50, Q75 |
| Age groups | N (%)  <18, 18–29 years, 30–39 years, 40–49 years, 50–59 years, ≥60 years |
| Sex  The sex distribution is given as a total for the whole period (2014-30 September 2023) and separately for each year. | female, male |
| Insurance status | member, family member, pensioner |
| Number of dengue disease periods treated in a university hospital/not in a university hospital | N (%) |

**Supplementary Table 2: Complications and risk factors of interest**

| **Type of complication/risk factor** | **Complication/risk factor** | **Acute or chronic** |
| --- | --- | --- |
| Hemorrhagic shock (other presentations of shock) |  | Acute |
| Multiple organ failure |  | Acute |
| Blood borne illnesses | Hepatitis B | Chronic |
|  | Hepatitis C | Chronic |
|  | HIV | Chronic |
| Anemia |  | Acute |
| Cardiac diseases | Myocardial infarction | Acute |
|  | Atrial fibrillation | Chronic |
|  | Ischemic heart disease | Chronic |
|  | Heart failure | Chronic |
| Diabetes | Type 1 Diabetes | Chronic |
|  | Type 1 Diabetes | Chronic |
| Gastrointestinal diseases | Gastroenteritis or colitis | Acute |
|  | Calculus of gallbladder or bile duct | Acute |
| Liver Failure | Acute or end stage liver disease | Acute or chronic |
| Respiratory | Acute respiratory distress syndrome | Acute |
| Renal diseases | Acute renal failure | Acute |
|  | Chronic kidney disease | Chronic |
| Neurological diseases | Stroke | Acute |
|  | Encephalopathy | Chronic |

**Supplementary Table 3: Hospitalization**

| **Endpoint** | **Output** |
| --- | --- |
| **Hospitalization**  (for all hospitalizations and only hospitalizations associated with dengue disease – main/secondary diagnosis=dengue) |  |
| Number of hospital stays | N patients with ≥1 hospital stay, N hospital stays total, Ø per patient, SD, Min, Max, Q25, Q50, Q75 |
| Length of stay in days (per hospital stay) | Ø length of stay per hospital stay, SD, Min, Max, Q25, Q50, Q75 |
| ICU treatment | N (%) (patients with ICU treatment) |

**Supplementary Table 4: Sick leave days**

| **Endpoint** | **Output** |
| --- | --- |
| Sick leave days |  |
| Number of patients with at least 1 sick day  N (%) | N (%) |
| Number of sick days (total) N | N |
| Sick days | Ø days per dengue disease period, SD, Min, Max, Q25, Q50, Q75 |
| Number of patients 16 – 65 years to determine the proportion of patients still of working age | N (%) |

**Supplementary Table 5: Costs**

| **Endpoint** | **Output** |
| --- | --- |
| **Costs** | Total costs (€), N total, N with costs =0, N with costs >0, Ø costs per dengue disease period, SD, Min, Max, Q25, Q50, Q75 |
| Total costs |  |
| Inpatient costs (direct costs) |  |
| Outpatient costs (direct costs) |  |
| Medication costs (direct costs) |  |
| Costs aids and remedies (direct costs) |  |
| Other costs |  |
| Costs of inpatient dengue disease periods with a severe course | Total costs (€), N total, N with costs =0), Ø costs per dengue disease period, SD, Min, Max, Q25, Q50, Q75 |

**Supplementary Table 6: Mortality**

| **Endpoint** | **Output** |
| --- | --- |
| Mortality | Total number of deceased patients during the index/follow-up of 3 months N (%),  (stratified by sex),  Average time to death (time between the middle of the index quarter and death in days) Ø SD, Min, Max, Q25, Q50, Q75, comorbidities of deceased |

.
