# Supplementary figures and images for: Assessment of the healthcare burden of dengue disease in Germany: a retrospective analysis of statutory health insurance data (2014–23)
Source: J Travel Med. 2026 Jun 11;33(5):taag047. doi: 10.1093/jtm/taag047 (PMC13318845; doi:10.1093/jtm/taag047)

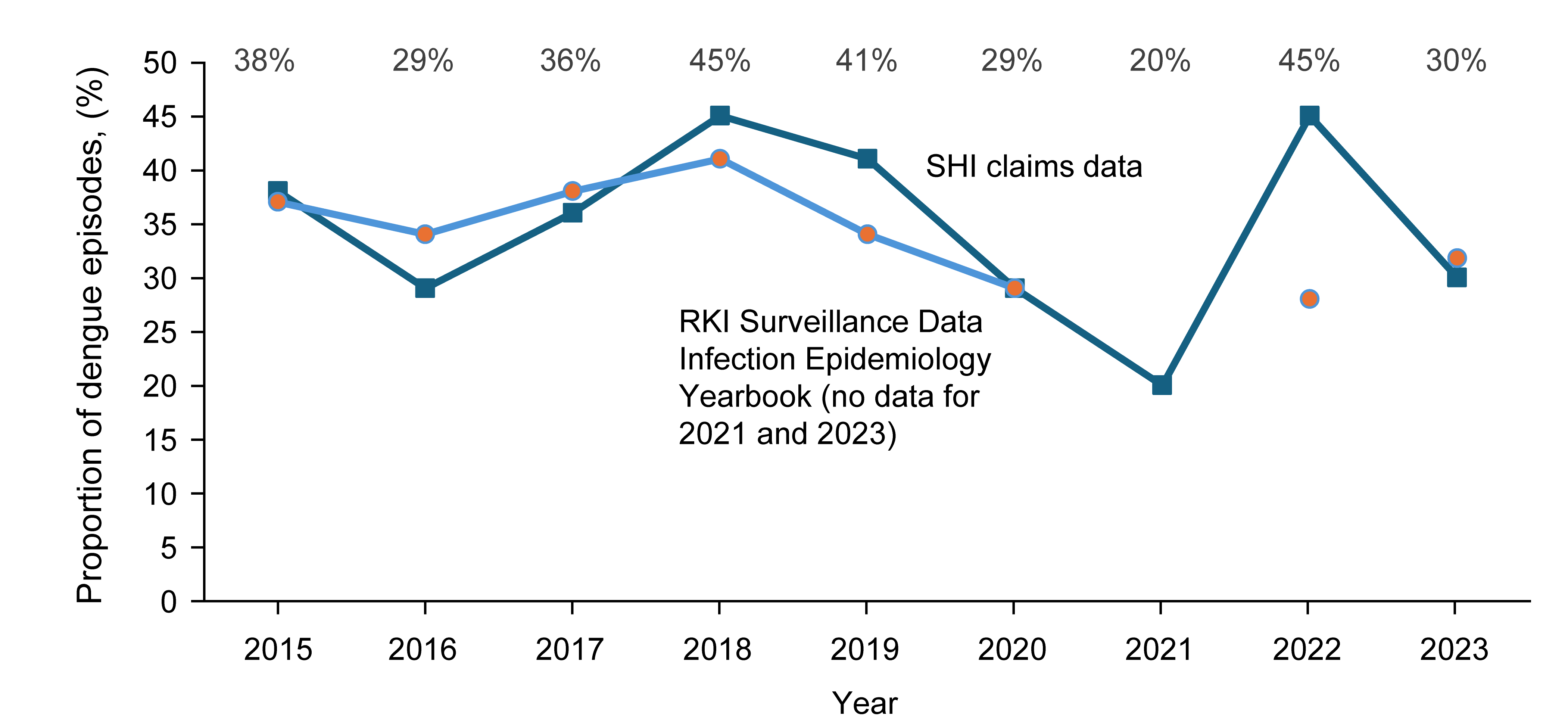

Supplement: Supplementary_material_taag047 [file supplementary_material_taag047.zip › TAPHI-28485 Vaccines BoD in Germany ms SUPP FIGURES_BF05_Figure S1.tif]

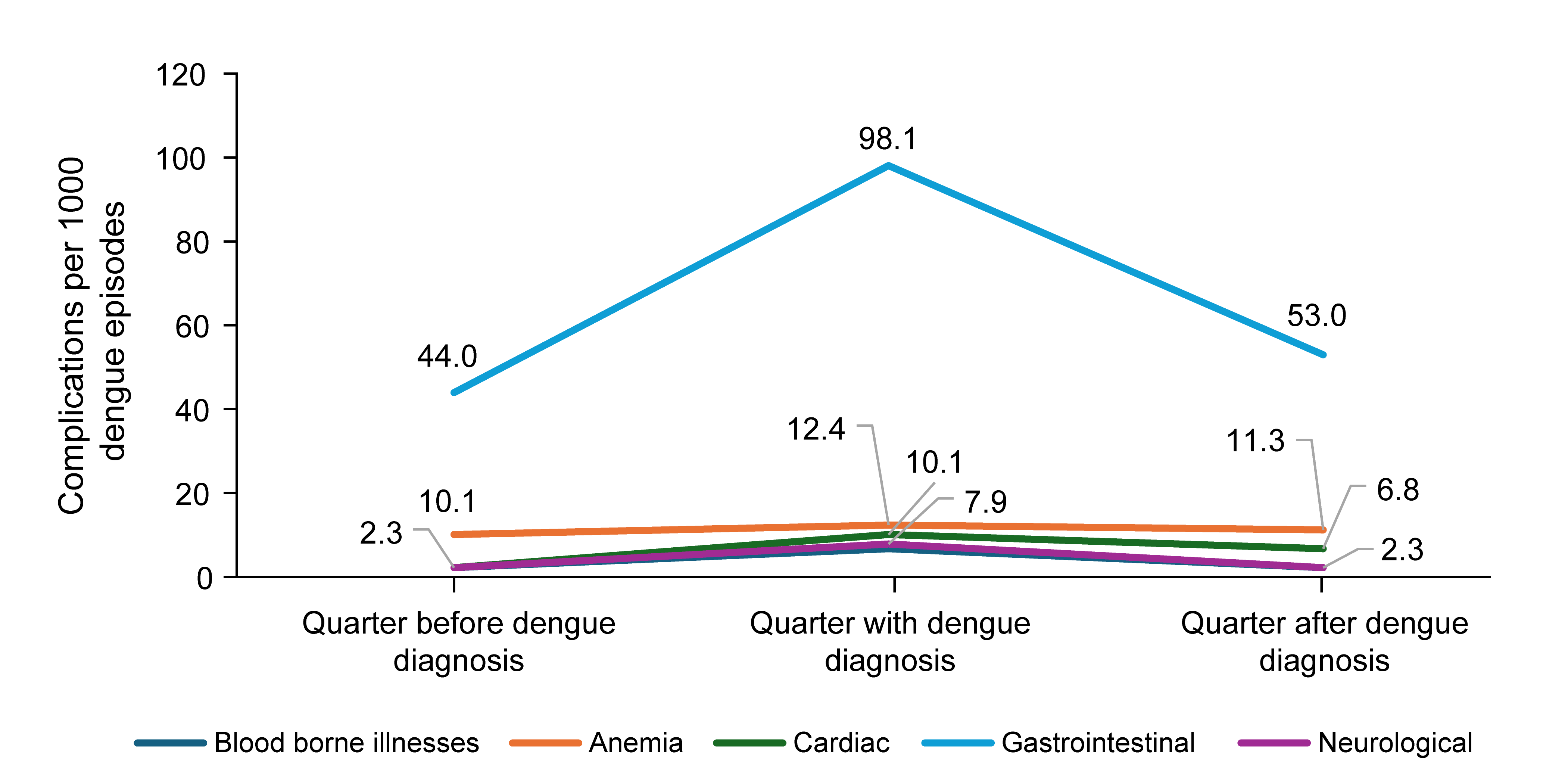

Supplement: Supplementary_material_taag047 [file supplementary_material_taag047.zip › TAPHI-28485 Vaccines BoD in Germany ms SUPP FIGURES_BF05_Figure S2.tif]

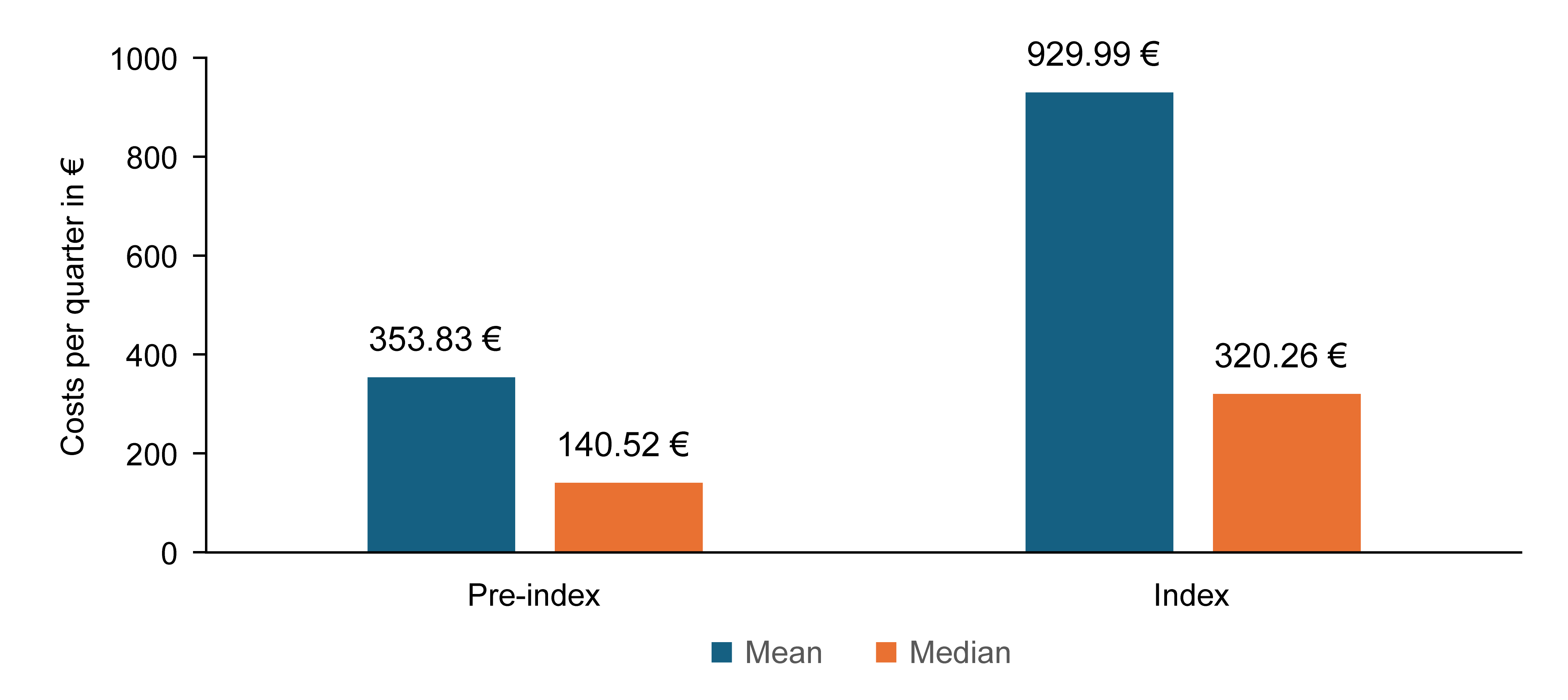

Supplement: Supplementary_material_taag047 [file supplementary_material_taag047.zip › TAPHI-28485 Vaccines BoD in Germany ms SUPP FIGURES_BF05_Figure S3.tif]

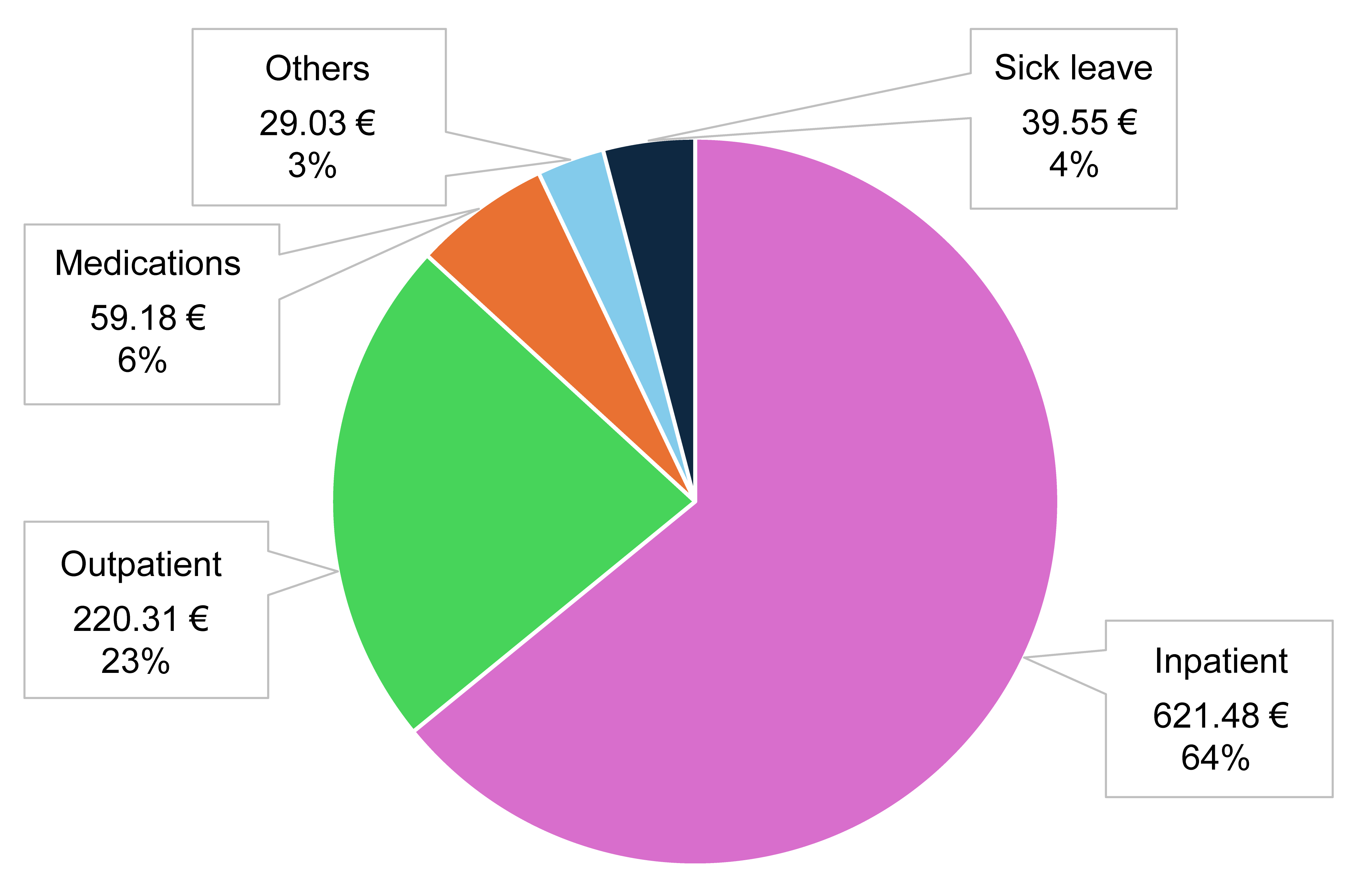

Supplement: Supplementary_material_taag047 [file supplementary_material_taag047.zip › TAPHI-28485 Vaccines BoD in Germany ms SUPP FIGURES_BF05_Figure S4.tif]

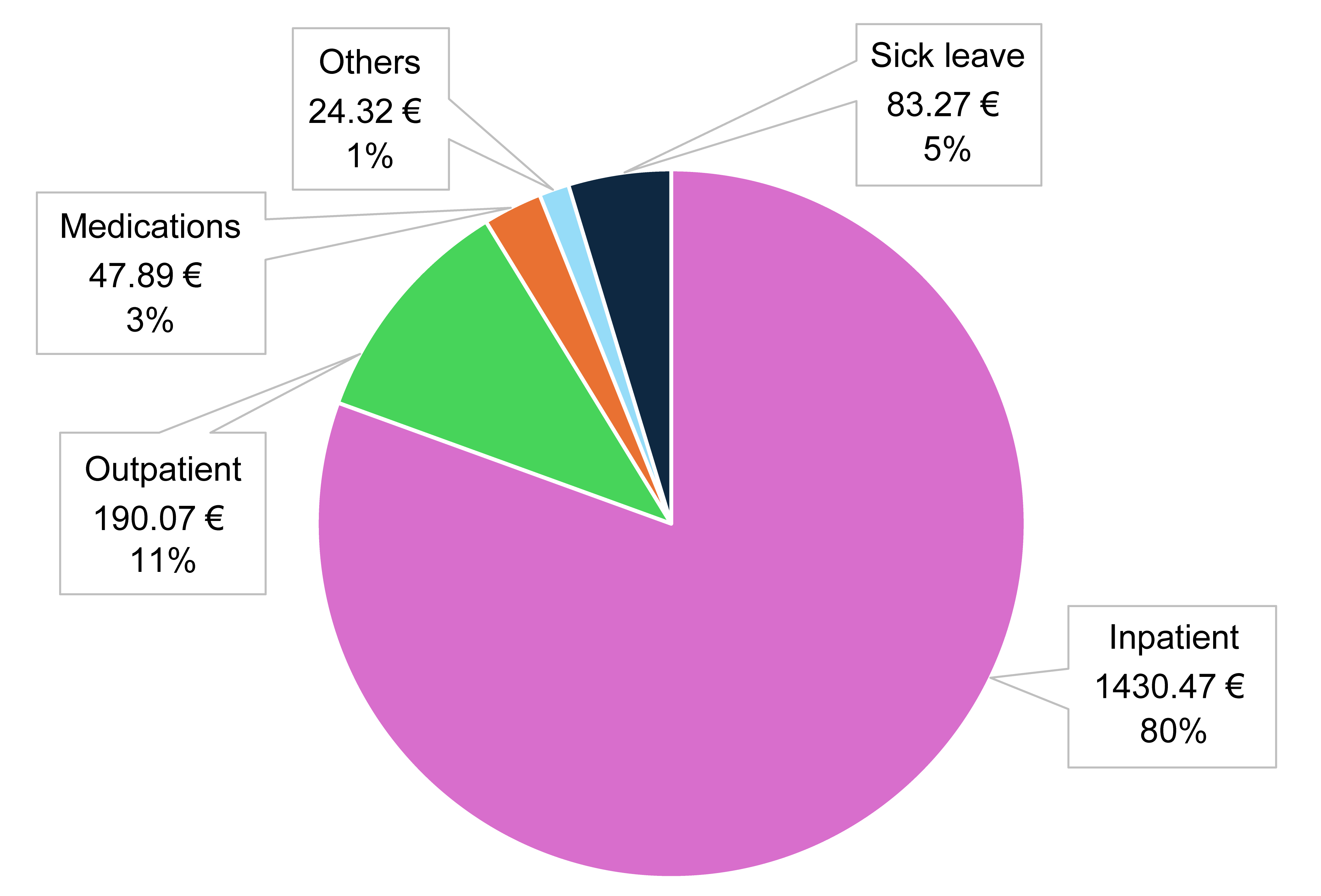

Supplement: Supplementary_material_taag047 [file supplementary_material_taag047.zip › TAPHI-28485 Vaccines BoD in Germany ms SUPP FIGURES_BF05_Figure S5.tif]
